# Characterization and phylogenetic analysis of the complete mitochondrial genome of *Conidiobolus* sp. (Entomophthorales: Ancylistaceae)

**DOI:** 10.1080/23802359.2019.1698340

**Published:** 2019-12-12

**Authors:** Xiaorong Sun, Dan Su, Wenjuan Gui, Feng Luo, Yue Chen

**Affiliations:** a Chengdu Bio-HT Company Limited, Chengdu, China;; b General Hospital of Ningxia Medical University, Yinchuan, China

**Keywords:** *Conidiobolus*, entomophthoroid fungus, mitochondrial genome, phylogenetic analysis

## Abstract

In the present study, we presented the complete mitochondrial genome of an entomophthoroid fungus *Conidiobolus* sp. The mitogenome of *Conidiobolus* sp. has a total length of 26,612 bp, with the base composition as follows: A (44.22%), T (27.10%), C (10.99%) and G (17.68%). The mitogenome contains 19 protein-coding genes, 2 ribosomal RNA genes (rRNA), and 23 transfer RNA (tRNA) genes. The taxonomic status of the *Conidiobolus* sp. mitogenome exhibited a close relationship with the mitogenome of *Conidiobolus heterosporus*.

*Conidiobolus*, a fungal genus in the family Ancylistaceae, is widely distributed in soil and among plants, insects, and amphibians (de Godoy et al. 2017). So far, 37 species have been described in this genus (Nie et al. 2018). *C. coronatus* causes conidiobolomycosis, a chronic granulomatous disease of submucosal and subcutaneous tissues (Moncada et al. 2016). Species of this genus have been widely used to study phylogenetic relationships of basal fungi (Nie et al. 2019). The complete mitogenome of *Conidiobolus* sp. will provide a reference for understanding the phylogeny and evolution of the basal fungi.

The specimen (*Conidiobolus* sp.) was isolated from soil in Guangyuan, Sichuan, China (104.41 E; 31.40 N) and was stored in Sichuan Academy of Agricultural Sciences Center for Culture Collection (No. Cps1). The total genomic DNA of *Conidiobolus* sp. was extracted using Fungal DNA Kit D3390-00 (Omega Bio-Tek, Norcross, GA, USA) and purified through a Gel Extraction Kit (Omega Bio-Tek, Norcross, GA, USA). Purified genomic DNA was also stored in the Sichuan Academy of Agricultural Sciences Center for Culture Collection (No. DNA_Cps1). We constructed the sequencing libraries with purified genomic DNA following the instructions of NEBNext^®^ Ultra™ II DNA Library Prep Kit (NEB, Beijing, China). Whole genomic sequencing (WGS) was performed by the Illumina HiSeq 2500 Platform (Illumina, SanDiego, CA, USA). The raw data obtained was first passed through a series of quality control steps (Li et al. 2018a). The complete mitogenome was *de novo* assembled using the clean data as implemented by SPAdes 3.9.0 (Bankevich et al. 2012). Gaps among contigs were filled by using MITObim V1.9 (Hahn et al. 2013). The obtained mitogenome was annotated using the MFannot tool (http://megasun.bch.umontreal.ca/cgi-bin/mfannot/mfannotInterface.pl), combined with manual corrections. tRNAs were annotated by tRNAscan-SE (Lowe and Eddy 1997).

The total length of *Conidiobolus* sp. circular mitogenome is 26,612 bp. This mitogenome was submitted to GenBank database under accession No. MN640580. The circular mitogenome contains 19 protein-coding genes, 2 ribosomal RNA genes (*rns* and *rnl*), and 23 transfer RNA (tRNA) genes. The base composition of the genome is as follows: A (44.22%), T (27.10%), C (10.99%) and G (17.68%).

To validate the phylogenetic position of *Conidiobolus* sp., we constructed phylogenetic trees of 12 closely related species. Bayesian analysis (BI) was used to construct the phylogenetic trees with the combined 14 core protein-coding genes according to Li (2018b, 2018c). As shown in the phylogenetic tree (Figure 1), the taxonomic status of the *Conidiobolus* sp. mitogenome exhibited a close relationship with the mitogenome of *C. heterosporus* (Nie et al. 2019).

**Figure 1.**
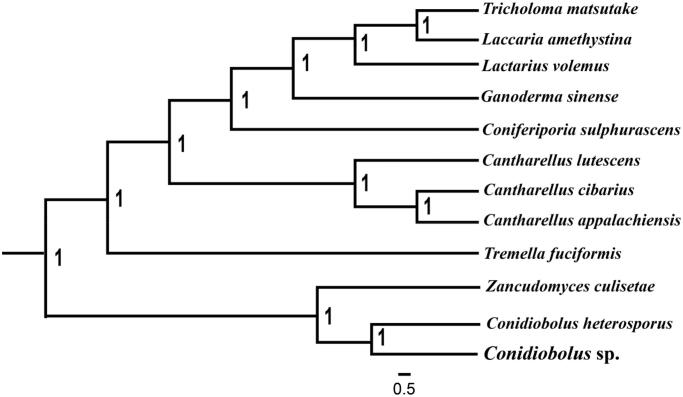
Molecular phylogenies of 12 species based on Bayesian inference analysis of the combined mitochondrial gene set (14 core protein-coding genes). Node support values are Bayesian posterior probabilities (BPP). Mitogenome accession numbers used in this phylogeny analysis: *Tricholoma matsutake* (JX985789), *Laccaria amethystina* (MK697669), *Lactarius volemus* (MH319474), *Ganoderma sinense* (KF673550), *Coniferiporia sulphurascens* (MK623260), *Cantharellus appalachiensis* (MG602716), *Cantharellus cibarius* (KC573037), *Cantharellus lutescens* (MG602719), *Tremella fuciformis* (MF422647), *Zancudomyces culisetae* (AY863213), *Conidiobolus heterosporus* (MK049352).
